# Analogies Between Platelet-Rich Plasma Versus Hyaluronic Acid Intra-articular Injections in the Treatment of Advanced Knee Arthritis: A Single-Center Study

**DOI:** 10.7759/cureus.61163

**Published:** 2024-05-27

**Authors:** Ahmed Abu-Awwad, Cristina Tudoran, Simona-Alina Abu-Awwad, Mariana Tudoran, Florica Voita-Mekeres, Cosmin Faur, Gheorghe Szilagyi

**Affiliations:** 1 Orthopedics and Traumatology, Department XV-Discipline of Orthopedics-Traumatology, Victor Babes University of Medicine and Pharmacy Timisoara, Timisoara, ROU; 2 Orthopedics and Traumatology, "Pius Brinzeu" County Emergency Hospital, Timisoara, ROU; 3 Orthopedics and Traumatology, Research Center University Professor Doctor Teodor Șora, Victor Babes University of Medicine and Pharmacy, Timisoara, ROU; 4 Department VII, Internal Medicine II, Discipline of Cardiology, Victor Babes University of Medicine and Pharmacy Timisoara, Timisoara, ROU; 5 Cardiology, Center of Molecular Research in Nephrology and Vascular Disease, Victor Babes University of Medicine and Pharmacy Timisoara, Timisoara, ROU; 6 Cardiology, "Pius Brinzeu" County Emergency Hospital, Timisoara, ROU; 7 Gynecology, “Pius Brinzeu” County Emergency Hospital, Timisoara, ROU; 8 Gynecology, Department XII-Discipline of Obstetrics and Gynecology, Victor Babes University of Medicine and Pharmacy Timisoara, Timisoara, ROU; 9 Cardiology, “Pius Brinzeu” County Emergency Hospital, Timisoara, ROU; 10 Morphological Disciplines, Faculty of Medicine and Pharmacy, University of Oradea, Oradea, ROU; 11 Orthopedics and Traumatology, “Pius Brinzeu” County Emergency Hospital, Timisoara, ROU; 12 Surgical Disciplines, Faculty of Medicine and Pharmacy, University of Oradea, Oradea, ROU

**Keywords:** functional outcomes, intra-articular injections, evidence-based medicine, regenerative medicine, quality of life

## Abstract

Background

Knee osteoarthritis (KOA), a degenerative joint disease, is a common cause of chronic knee pain and disability in adults. Conservative management options are the first-line approach, but intra-articular injections, such as platelet-rich plasma (PRP) and hyaluronic acid (HA), are considered for advanced cases. This study aims to compare the efficacy of PRP versus HA injections in patients with advanced KOA.

Methods

A retrospective study was conducted on 145 patients with advanced KOA. Seventy patients received PRP injections, while 75 patients received HA injections. The Visual Analog Scale (VAS), Western Ontario and McMaster Universities Osteoarthritis Index (WOMAC) score, and International Knee Documentation Committee (IKDC) score were employed to evaluate the treatment's efficacy. Adverse events associated with these injections were also recorded.

Results

Both PRP and HA injections significantly reduced pain and improved joint function in patients with advanced KOA. PRP injections were slightly more effective than HA injections in reducing pain scores. Both treatments showed similar improvements in functional outcomes. Adverse events were minimal and self-limiting for both treatments.

Conclusions

Both PRP and HA injections effectively ameliorate advanced KOA by reducing pain and improving function. PRP injections showed a slightly greater improvement in pain scores and functional outcomes. The choice between PRP and HA injections may depend on factors like cost, availability, and patient preference. Further research is needed to validate these findings and understand treatment suitability for different patient populations.

## Introduction

Knee osteoarthritis (KOA) is a pervasive degenerative joint disease that is typified by gradual articular cartilage loss, synovial inflammation, and joint space diminution. As it is the principal cause of chronic knee pain and disability in adults over 50 years, KOA significantly impacts patients' quality of life (QOL) [1,2,3]. KOA, often regarded as a degenerative knee joint disease, is globally recognized for its debilitating impact on millions of lives. KOA, characterized by the steady degeneration of joint structures (the infrapatellar fat pad, subchondral bone, and meniscus), is a comprehensive disease that affects the entire joint complex [1,2,4]. Specifically, in KOA, the infrapatellar fat pad, a vital structural component beneath the kneecap responsible for joint biomechanics, experiences detrimental changes that lead to inflammation, thus contributing to pain and functional impairment. Similarly, subchondral bone, located beneath the cartilage, undergoes remodeling, altering its microstructure and composition, thereby impacting joint stability and propelling KOA progression [4,5]. The meniscus, another key structure within the knee joint, can become damaged, further limiting joint mobility and exacerbating joint stress [6]. A profound understanding of these complex mechanisms and interactions within joint tissues is indispensable for the evolution of KOA diagnostic and treatment strategies. By addressing this disease as a whole-joint ailment and acknowledging the interplay among the infrapatellar fat pad, subchondral bone, and meniscus, we can propel advancements in managing KOA, thereby improving patient outcomes [7-10].

Therapeutic strategies have historically encompassed conservative management involving exercise, physiotherapy, and analgesics, with intra-articular injections such as platelet-rich plasma (PRP) and hyaluronic acid (HA) emerging as alternatives when traditional approaches failed [11-13].

While several treatment options, such as physical therapy, medication, and surgery, are available for patients with advanced KOA, non-surgical alternatives like PRP and HA injections have garnered increasing attention [13,14]. PRP injections, using a patient's own blood, provide a concentrated solution of platelets and growth factors, promoting healing and reducing inflammation. On the other hand, HA injections deliver hyaluronic acid, a natural joint lubricant and shock absorber, directly into the affected joint [1,2,11,13,15]. Both PRP and HA injections have demonstrated efficacy in managing KOA symptoms. However, the superior treatment between the two for advanced KOA remains unclear. Opinions concerning the efficacy and benefits of these therapeutic options are debated in several studies [15-17].

This study aims to compare the effectiveness of PRP and HA injections on pain reduction and functional improvement in patients with advanced KOA to determine the best therapeutic option for ameliorating their symptoms until knee-replacement procedures become unavoidable. Through this comparative analysis, we aim to provide patients and clinicians with a comprehensive understanding of the potential benefits and risks associated with each treatment, facilitating informed decisions on treatment options.

## Materials and methods

Study population

The study was conducted at the “Pius Brinzeu” County Emergency Hospital, Timisoara, Romania. This study, conducted prospectively from January 1, 2022, to June 30, 2023, included 145 patients, aged between 45 and 75 years, diagnosed with advanced KOA, who were treated on a randomized basis (75/75) either with platelet-rich plasma (PRP) or with hyaluronic acid (HA) injections. Of the PRP subgroup, after being included in our study, five subjects declined their participation, so only 70 patients remained to complete the whole study.

The study population was stratified into two cohorts: Group 1, comprising 70 patients who received PRP injections, and Group 2, including 75 patients who underwent HA injections.

The inclusion criteria for this study were as follows: ambulatory patients, aged between 45 and 75 years, already diagnosed with grade III or IV KOA confirmed through imaging investigations, without any prior surgical treatment of the affected joint, excepting anti-inflammatory and/or pain relief medication, and without a previous history of PRP or HA injections. They also agreed to participate in our study and to sign an informed consent.

Exclusion criteria were established to minimize confounding effects and included individuals with severe systemic diseases (e.g., uncontrolled diabetes, autoimmune disorders, malignancies, cardiovascular diseases requiring anticoagulant or high doses of antiaggregant therapy) [18], recent intra-articular therapy within three months preceding the study, active infections, known allergies or hypersensitivity to PRP or HA, knee joint instability or ligamentous laxity, obesity (body mass index > 30), and those who had received treatment outside the study time frame or at different healthcare facilities.

**Figure 1:**
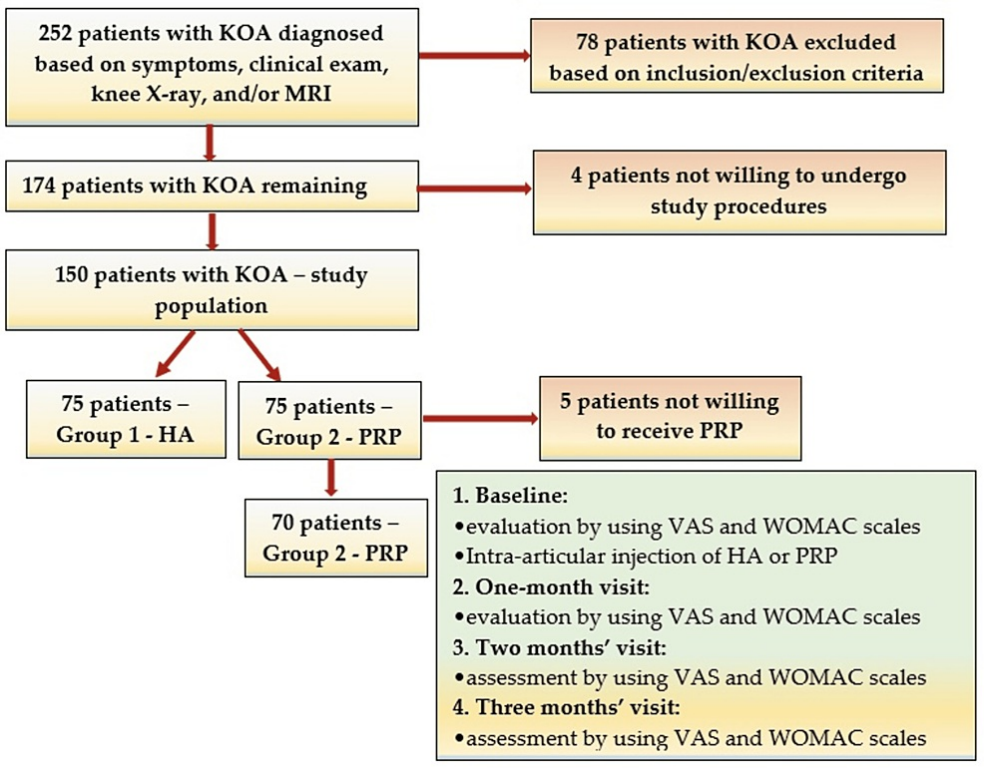
Flow chart describing the selection process and study procedures KOA: knee osteoarthritis; VAS: Visual Analog Scale; WOMAC: Western Ontario and McMaster Universities Osteoarthritis Index; PRP: platelet-rich plasma; HA: hyaluronic acid

Treatment outcomes were assessed using standardized tools, such as the Visual Analog Scale (VAS) and the Western Ontario and McMaster Universities Osteoarthritis Index (WOMAC) questionnaires [10,19]. The selection process and study procedures are illustrated in the flow chart depicted in Figure 1.

The Visual Analog Scale (VAS) is a simple yet effective measurement instrument designed to document the intensity of pain experienced by patients, allowing a subjective comparison of pain levels pre- and post-treatment. In its traditional form, it is a 10 cm line with endpoints defining extreme limits such as 'no pain at all' and 'pain as bad as it could be'. The patient is asked to mark his or her clinical pain level on this line between the two endpoints. This assessment was an important instrument in evaluating the success of the PRP and HA injections in mitigating the pain associated with KOA [10].

The WOMAC questionnaire offers a more comprehensive evaluation. Typically administered as a self-report questionnaire, the respondents rate their experiences on a Likert scale, with responses ranging from none to extreme, or on a numerical scale (from 0 to 4). It is divided into three subscales, referring to pain, stiffness, and physical function, and contains a series of questions directed to examine these symptoms in individuals with KOA. Researchers and healthcare professionals can analyze the scores from each subscale individually or combine them to obtain an overall assessment of the individual's KOA-related health status. The WOMAC scale has become a widely accepted and validated tool for assessing the impact of osteoarthritis on individuals, and its use is prevalent in both clinical practice and research settings. Higher scores on the WOMAC subscales indicated greater pain, stiffness, or functional impairment [10,19,20].

VAS scores were documented at baseline, then at one month, two months, and three months post-injections, while WOMAC scores were recorded at baseline, at one, at two, and at the three-month mark. Adverse events related to the treatments were also monitored and noted. The obtained data from these assessments were subjected to statistical analysis to identify significant changes and draw objective comparisons between the two treatment modalities. This comprehensive evaluation process provided valuable insights into the overall effectiveness of PRP and HA injections in managing pain, alleviating stiffness, and improving functional abilities in patients with advanced KOA. This methodical approach, including the establishment of clear inclusion and exclusion criteria, allowed the collection of relevant data while ensuring homogeneity and consistency in the studied population.

The study was conducted in accordance with the Declaration of Helsinki and approved by the Ethics Committee of the “Pius Brinzeu” County Emergency Hospital (396/15.05.2023).

Therapeutic methods

The chosen therapeutic methods employed for this study encompassed the utilization of either HA or PRP injections. The HA injections utilized a composition containing 16 mg/ml of hyaluronic acid, 35 mg/ml of mannitol, sodium chloride, and water for the injectable solution. This injectable solution is designed to emulate the properties of natural synovial fluid, thereby providing lubrication and shock absorption to the arthritic joint.

For PRP injections, a standardized protocol was adhered to, involving the centrifugation of autologous blood. Blood was drawn from the patient's vein, placed into a test tube, and centrifuged at 3000 revolutions per minute for 10 minutes. The resultant plasma, enriched with platelets and growth factors, was then injected into the knee joint. PRP leverages the body's intrinsic healing mechanisms, potentially promoting tissue regeneration and symptom alleviation.

Statistical analysis

The dataset generated for this investigation was subjected to rigorous analysis using the GraphPad Prism software (version 5).

Descriptive statistics were used to summarize the demographic characteristics of the study population. To establish the sample size, we employed the Cohen h effect size calculation for the total group of 145 patients, with a significance level (α) of 0.05 and an expected proportion of 0.55 for the PRP group (p1) and 0.5 for the HA group (p2). The results confirmed that our sample size is sufficient to draw significant conclusions. To assess the comparability of the two groups, statistical tests were conducted. The independent samples t-test was used to analyze the mean age of the patients, and the chi-square test or Fisher's exact test (if cell counts were small) was employed to examine the differences between the clinical characteristics in the two groups. The t-test, by comparing the group means, was able to ascertain if the observed differences were of genuine statistical significance or if they could be ascribed to random variations within the data. The results were then expressed as the mean value plus or minus the standard deviation (SD). The threshold for statistical significance was set at p-values less than 0.05.

For the evaluation of treatment effectiveness, the changes over time were analyzed using statistical tests. The paired samples t-test or Wilcoxon signed-rank test (for non-parametric data) was performed to examine the significance of changes concerning the VAS and WOMAC scores within each treatment group at one month, two months, and three months after the injections.

## Results

Our study enrolled a total of 145 patients with advanced KOA. Of these patients, 70 received PRP injections, and 75 received HA injections. The demographic and clinical characteristics of the patients are presented in Table 1.

**Table 1:** Demographic and clinical characteristics of patients receiving PRP and HA injections PRP: platelet-rich plasma; HA: hyaluronic acid

Characteristic	PRP Group (n=70)	HA Group (n=75)	P-value t-test
Age (years)	62.1 ± 8.9	63.4 ± 9.6	0.4001
Gender (men/women)	27/43	31/44	M/F <0.001
Body mass index (kg/m^2)	29.4 ± 4.1	29.8 ± 4.2	0.5630
Duration of symptoms (months)	28.9 ± 7.2	29.5 ± 8.1	0.6390
Kellgren-Lawrence grade (I/II/III/IV)	0/0/58/12	0/0/55/20	-
Previous knee surgery (Yes/No)	no	no	-

The mean age and standard deviation (±) were calculated for both groups: 62.1 ± 8.9 years for the PRP group and 63.4 ± 9.6 years for the HA group, and differences were analyzed by using an independent sample t-test. In the PRP group, the mean age of the patients was 62.1 ± 8.9 years, while in the HA group, it was 63.4 ± 9.6 years. There were no significant differences between the PRP and HA groups concerning age (p=0.4001). Although the female gender prevailed in both groups (p<0.001), they were comparable in terms of age, gender, and baseline characteristics. The chi-square test or Fisher's exact test (if cell counts were small) was employed to examine the differences in gender, body mass index, duration of symptoms, Kellgren-Lawrence grade, and previous knee surgery between the two groups. The results indicated no significant differences in gender, body mass index (p=0.5630), or duration of symptoms (p=0.6390) between the PRP and HA groups. However, there were slightly more patients with Kellgren-Lawrence grades III and IV in the PRP group compared to the HA group (Table 2). Additionally, a slightly higher number of patients in both groups had previous knee surgery. Overall, the two groups were well-matched in terms of their demographic and clinical characteristics, allowing for a valid comparison of the effectiveness of PRP and HA injections in the management of advanced KOA.

**Table 2:** Results of HA vs. PRP SD: standard deviation; BMI: body mass index; PRP: platelet-rich plasma; HA: hyaluronic acid

Treatment group	Number of patients	Mean age ± SD	Gender (No. of males/females)	BMI	Duration of symptoms	Kellgren-Lawrence grade III and IV	Previous knee surgery
PRP	70	62.1 ± 8.9	Males: 27 / Females: 43	29.4 ± 4.1	28.9 ± 7.2	III – 58 IV - 12	No
HA	75	63.4 ± 9.6	Males: 31 / Females: 44	29.8 ± 4.2	28.5 ± 8.1	III – 55 IV - 20	No

Functional improvement

Both PRP and HA injections were also effective in improving joint function in patients with advanced KOA. However, the results showed that PRP injections were more effective than HA injections in improving functional scores. This finding suggests that PRP injections may be more beneficial for patients who are experiencing a significant decrease in function due to their condition.

**Figure 2:**
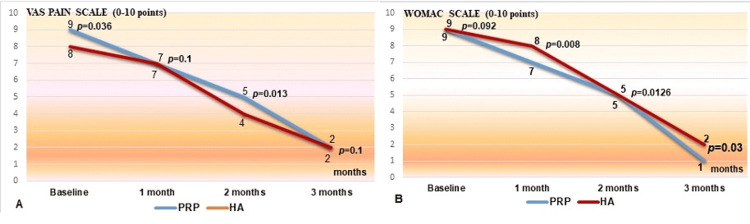
Comparative analysis of pain intensity reduction following PRP and HA treatments A: Three-month follow-up by employing Visual Analog Scale (VAS); B: Western Ontario and McMaster Universities Osteoarthritis Index (WOMAC) scale PRP: platelet-rich plasma; HA: hyaluronic acid

The VAS scores significantly improved in both groups at one month and at three months after the injections (p=0.1) (Figure 2A). However, there was no significant difference in VAS scores between the two groups at any time point (p>0.05). The WOMAC scores significantly improved in both groups at three months after the injections (p=0.03) (Figure 2B).

For the evaluation of treatment effectiveness, the changes in VAS scores and WOMAC scores over time were analyzed using statistical tests. The paired samples t-test or Wilcoxon signed-rank test (for non-parametric data) was performed to examine the significance of changes within each treatment group at one month, two months, and three months after the injections.

Although our results revealed significant improvements in VAS scores in both the PRP and HA groups, there was no significant difference in VAS scores between the two groups at all time points (p=0.1 at one month, p=0.013 at two months, and p=0.1 at three months, Figure 2A). Similarly, the WOMAC scores significantly improved in both groups at three months after the injections (p=0.008 at one month, p=0.01 at two months, and p=0.03 at three months, Figure 2B). The mechanism behind this improvement may be due to the regenerative properties of the growth factors present in PRP, which promote tissue repair and regeneration in the joint.

The statistical analysis demonstrated the effectiveness of both PRP and HA injections in improving joint function, as indicated by the significant improvements in VAS scores and WOMAC scores.

However, PRP injections were found to be more effective than HA injections in improving functional scores (but not statistically significant), suggesting their potential benefits for patients experiencing a significant decrease in function. The regenerative properties of growth factors present in PRP may contribute to this improvement by promoting tissue repair and regeneration in the joint.

Adverse events

In regard to adverse events, both PRP and HA injections demonstrated a favorable safety profile. The occurrence of adverse events was relatively low, with the primary complications comprising local oedema (observed in 19 patients), minor irritation post-infiltration (reported by six patients), and pain that persists for two to three days post-infiltration (observed in three patients). The minimal disparity in adverse events between the two treatments suggests their comparable safety in the management of advanced knee arthritis symptoms.

However, it is crucial to underscore that despite the generally benign nature of these procedures, some patients might experience complications such as pain, swelling, or infection at the injection site. Therefore, vigilant monitoring post-injection is integral to ensuring patient safety and promptly addressing any complications.

## Discussion

The objective of the current investigation was to evaluate and compare the efficacy of PRP injections and HA injections in managing advanced KOA, deploying a randomized controlled trial methodology for the rigorous assessment of these treatment alternatives.

The outcomes of the study substantiated that both PRP and HA injections significantly ameliorated pain relief and joint function in patients diagnosed with advanced KOA. However, a comparative analysis between the two treatment modalities demonstrated variations in efficacy. For the dimension of pain relief, the evidence suggests that PRP injections displayed superior effectiveness relative to HA injections, with patients receiving PRP therapy reporting a more significant diminution in pain scores. This data corroborates prior research, such as the one by Patel et al. [17], underscoring the regenerative properties of PRP and its capacity to attenuate pain through the stimulation of tissue repair and inflammation mitigation in comparison to other observations concerning therapy with HA [21,22] or a meta-analysis concerning intra-articular therapies in KOA [23].

In terms of improving joint function, both PRP and HA therapy manifested positive effects. Nevertheless, no substantial differences were discernible between the two treatment groups concerning functional outcomes, implying that both PRP and HA may contribute to the restoration of joint function in advanced KOA, albeit PRP could offer a distinct advantage in terms of pain alleviation [23-25].

The long-term implications of PRP and HA injections were also subject to exploration. Although the primary focus was on short-term efficacy, contemplating the potential sustainability of the treatments is essential. Existing research has proposed that the advantageous effects of PRP injections could persist for an extended duration in comparison to HA injections. Future research endeavors should investigate long-term patient follow-ups to evaluate the durability of the observed benefits and monitor the disease trajectory over time.

Consideration of safety profiles and adverse effects is crucial when contrasting treatment modalities. Both PRP and HA therapies exhibited good tolerance in this study, with no significant adverse events reported. Nevertheless, comprehensive assessments of the safety profiles of these interventions necessitate further investigations with larger cohorts and extended follow-up periods [25,26].

The decision to opt for PRP or HA injections should not hinge solely on efficacy. Other variables, such as cost, accessibility, patient preferences, and practitioner expertise, should factor into treatment decisions. Engaging in shared decision-making between patients and healthcare professionals is vital for optimizing treatment outcomes and addressing individualized patient needs [27-29].

The principal outcomes of interest in this study were quantitatively evaluated using two widely accepted scoring systems: the Visual Analog Scale (VAS) and the Western Ontario and McMaster Universities Osteoarthritis Index (WOMAC). These instruments were chosen as they each provide a comprehensive and multi-faceted assessment of the disease's impact on the participants' quality of life.

Concurrently, the Western Ontario and McMaster Universities Osteoarthritis Index (WOMAC) was employed to further appraise the disease's impact on the patients' lives.

In order to better discern the independent effect of PRP and HA injections on these outcomes, a multivariate analysis was conducted., and its results evidenced a significant revelation: there was a noticeable improvement in both VAS and WOMAC scores following treatment with both PRP and HA injections. This improvement was found to be independent of other variables considered in the study, providing robust evidence for the efficacy of these treatments in alleviating pain and improving function in patients suffering from knee osteoarthritis [4].

In summary, this investigation compared the effectiveness of PRP versus HA injections in treating advanced KOA and found that both treatments facilitated improvements in pain relief and joint function. PRP injections demonstrated a superior pain reduction effect, while both treatments yielded similar functional outcomes. This suggests that PRP injections may be more beneficial for patients who are experiencing a significant decrease in function due to their condition; thus, increasing effort tolerance may reduce the risk of deep vein thrombosis and pulmonary embolism [30].

There were no significant differences in adverse events between the two treatments, with both PRP and HA injections being generally well-tolerated by patients. This suggests that both treatments are safe options for managing the symptoms of advanced knee arthritis until knee-replacement procedures and their consequences become unavoidable [31]. Further research involving larger sample sizes, extended follow-up periods, and diverse patient populations is necessary to confirm these findings and yield more comprehensive insights into the comparative efficacy of PRP and HA injections in the management of advanced KOA.

Limitations

One important limitation is represented by the relatively small sample size, which could have affected the statistical power and the broader applicability of the findings. While smaller sample sizes can offer initial insights and guide further research, they may not always capture the full range of variability present in larger populations. Thus, the findings may not be wholly representative of the broader population affected by KOA.

Another limitation is the short follow-up period and the specific population of advanced KOA patients, which in a single orthopedic clinic limits the generalizability of the results to other stages of the disease or different patient populations from various geographic areas. Furthermore, the follow-up period was relatively short, which may have limited the ability of the study to observe the long-term effects of PRP and HA injections on KOA. Understanding the enduring impacts of these treatments is essential for evaluating their sustained effectiveness and potential side effects.

Despite these limitations, the study provides valuable preliminary evidence supporting the efficacy of both PRP and HA injections as treatment modalities for individuals grappling with advanced KOA. These findings, while tentative, underscore the need for continued research in this area. Future studies with larger sample sizes and extended follow-up periods could substantiate and expand upon these initial observations. They could also delve deeper into the comparative efficacy of PRP and HA injections, potentially offering more robust and generalizable conclusions that could inform clinical practice guidelines for the management of KOA [5].

## Conclusions

Our investigation into the efficacy of PRP and HA injections for advanced KOA revealed that both treatments significantly reduced pain and improved joint function. While both treatments enhanced joint function, there were no notable differences between them in this respect, suggesting that each could be beneficial for ameliorating joint function in advanced KOA cases. Safety evaluations indicated that both PRP and HA were well tolerated by patients, with no significant adverse events reported. The choice between PRP and HA injections should consider factors beyond efficacy, including cost, patient preferences, and accessibility. Our findings support previous observations on the effectiveness of PRP and HA injections in managing advanced KOA symptoms, with PRP offering some advantages in pain management. 
